# ChatGPT Applications in Heart Failure: Patient Education, Readability Enhancement, and Clinical Utility

**DOI:** 10.3390/jcdd12110422

**Published:** 2025-10-24

**Authors:** Robert S. Doyle, Jack Hartnett, Hugo C. Temperley, Cian P. Murray, Ross Walsh, Jamie Walsh, John McCormick, Catherine McGorrian, Katie Murphy, Kenneth McDonald

**Affiliations:** 1 Cardiology Department, Mater Misericordiae University Hospital, Eccles St., D07 R2WY Dublin, Ireland; 2 Radiology Department, St. James’s Hospital, James’s St., Saint James, D08 NHY1 Dublin, Ireland; 3 Trinity College Dublin, The University of Dublin, College Green, D02 PN40 Dublin, Ireland; 4 Cardiology Department, St. James’s Hospital, James’s St., Saint James, D08 NHY1 Dublin, Ireland; 5 Cardiothoracic Surgery Department, Mater Misericordiae University Hospital, Eccles St., D07 R2WY Dublin, Ireland; 6 Cardiology Department, St. Vincent’s University Hospital, Elm Park, D04 T6F4 Dublin, Ireland

**Keywords:** ChatGPT, heart failure, artificial intelligence, patient education, systematic review, language models

## Abstract

**Background:** Heart failure (HF) affects over 64 million people globally, imposing substantial morbidity, mortality, and economic burdens. Despite advances in guideline-directed therapies, adherence remains suboptimal due to low health literacy and complex regimens. ChatGPT, an advanced large language model by OpenAI, offers conversational capabilities that could enhance HF education, management, and research. This systematic review synthesizes evidence on ChatGPT’s applications in HF, evaluating its accuracy in patient education and question-answering, enhancing readability, and clinical documentation/symptom extraction. **Methods:** Following PRISMA guidelines, we searched PubMed, Embase, and Cochrane up to July 2025 using the terms “ChatGPT” and “heart failure”. Inclusion: Studies on ChatGPT (3.5 or 4) in HF contexts, such as in education, readability and symptom extraction. Exclusion: Non-HF or non-ChatGPT AI. Data extraction covered design, objectives, methods, and outcomes. Thematic synthesis was applied. **Results:** From 59 records, 7 observational studies were included. Themes included patient education/question-answering (*n* = 5), readability enhancement (*n* = 2), and clinical documentation/symptom extraction (*n* = 1). Accuracy ranged 78–98%, with high reproducibility; readability improved to 6th–7th grade levels; and symptom extraction achieved up to 95% F1 score, outperforming traditional machine learning baselines. **Conclusions:** ChatGPT shows promise in HF care but requires further randomized validation for outcomes and bias mitigation.

## 1. Introduction

Heart failure (HF) is a growing public health concern, affecting approximately 7 million adults in the United States alone [1]. Although progress in HF treatment strategies has been made, the condition still exerts a major economic strain on the U.S. healthcare infrastructure, with expenses estimated at over $30 billion in 2012, and projected to more than double by 2030 [1]. The implementation of guideline-directed medical therapy (GDMT) alongside rigorous patient monitoring has notably decreased HF-associated death rates and hospital readmissions in the past few decades [2,3]. However, contemporary heart failure management is highly labour-intensive, both for patients and healthcare providers. Innovative approaches will be required to meet the increasing demand for heart failure care with finite healthcare resources [4].

Artificial intelligence (AI) and machine learning (ML) methods have been investigated as viable aids to address these issues [5]. ML approaches have proven advantageous when integrated with traditional statistical tools across multiple areas of cardiovascular care [6]. AI systems offer opportunities to advance HF management by facilitating informed clinical judgments, refining therapy distribution and identifying cases of undetected or progressing HF [7,8,9,10,11,12,13]. Furthermore, ML is being effectively used in the study of HF diagnostics via the examination of varied datasets compiled of electrocardiograms, echocardiograms, wearable sensors, and recorded heat sound auscultations [14,15].

In recent times, ChatGPT, an AI model developed by OpenAI using the Generative Pre-trained Transformer architecture, has captured global interest due to its proficiency in creating responses that closely resemble human dialogue. It stands as one of the most widely-used large language models available. Equipped to grasp and mirror the complexities of natural language, ChatGPT is swiftly gaining traction in medical environments. This model has exhibited capability in potentially supporting healthcare professionals with diagnostic reasoning and developing individualized care plans. However, the existing evidence on ChatGPT’s uses in heart failure is limited to a small number of observational studies demonstrating reasonable performance. To date, no randomized controlled trials have been conducted, and there is no documented evidence of its impact on reducing patient admissions or heart failure events. The aim of this systematic review is to critically appraise and synthesize the available evidence on ChatGPT in heart failure. Finally, we highlight the current ethical challenges in adopting ChatGPT technology.

## 2. Methods

### 2.1. Study Design and Reporting Guidelines

This study is a systematic review of original studies and follows the preferred reporting items for systematic reviews and meta-analyses (PRISMA) reporting guidelines. Our systematic review was registered on PROSPERO in September 2025 (ID: CRD420251141530).

### 2.2. Search Strategy

A comprehensive literature search was performed across three major electronic databases: PubMed, Embase, and Cochrane Library from their inception through July 2025, aiming to capture all relevant publications without temporal limitations. The search strategy employed a combination of controlled vocabulary (such as Medical Subject Headings [MeSH] terms where applicable) and free-text keywords to maximize sensitivity. Key search terms were structured as follows: (“ChatGPT” OR “GPT” OR “Generative Pre-trained Transformer”) AND (“heart failure” OR “congestive heart failure” OR “HF” OR “CHF”). Boolean operators (AND/OR) were used to refine and broaden the query, with truncation and wildcard symbols applied as needed to account for variations in terminology. No language restrictions were imposed to promote inclusivity and avoid potential bias from excluding non-English studies. Additionally, to identify any overlooked publications, the reference lists of all included studies, as well as pertinent narrative reviews and related articles, were manually hand-searched. Grey literature sources, including conference proceedings and preprint servers, were also screened for completeness.

### 2.3. Inclusion and Exclusion Criteria

The inclusion criteria were as follows:

Published studies demonstrating the current or future use and future potential of ChatGPT in heart failure

ChatGPT’s role was considered relevant if it referred to one of the following three domains: education and question-answering, readability enhancement and clinical applications.

Publications relating to both clinical use and academic use are eligible for inclusion.

Published in the English language.

The exclusion criteria were as follows:

Abstract-only publications.

Studies failing to discuss or denote ChatGPT in heart failure.

### 2.4. Study Selection, Data Extraction and Critical Appraisal

A database was created using EndNote X9 (The EndNote Team, Clarivate, Philadelphia, PA, USA, 2013). Abstracts were screened by two independent reviewers (RD and CM) based on inclusion/exclusion criteria, focusing on three domains: ChatGPT’s enhancement of patient education and management; factual accuracy of outputs, and comparison to clinical standards. Duplicates were removed, and discrepancies resolved through discussion with a third reviewer (HCT), excluding articles upon agreement. Full texts were evaluated by two reviewers for eligibility, with reference lists hand-searched for additional studies. Data extraction followed the PICOTS framework (Population, Intervention, Comparator, Outcomes, Timing, Setting) using Covidence (Covidence systematic review software, Veritas Health Innovation, Melbourne, VIC, Australia; https://www.covidence.org/, accessed 10 September 2025). Conflicts were resolved via discussion, with final decisions by the senior author.

A critical appraisal of the methodological quality and risk of bias of the included studies was not performed. There is currently no risk of bias (ROB) tool specific for ChatGPT and as there is no true population ROB cannot be applied to these studies.

### 2.5. Synthesis

A thematic synthesis approach was used to group study findings into three categories: education and question-answering, readability enhancement, and clinical applications. Readability enhancement was defined as the simplification and rephrasing of complex patient education materials to improve accessibility and comprehension while maintaining accuracy. It was measured using tools like the Flesch-Kincaid grade level scores, Patient Education Materials Assessment Tools (PEMAT) readability and actionability percentages, and assessments of word difficulty. Meta-analysis was not conducted due to significant heterogeneity in study designs, interventions, and outcome measures.

## 3. Results

**Figure 1:**
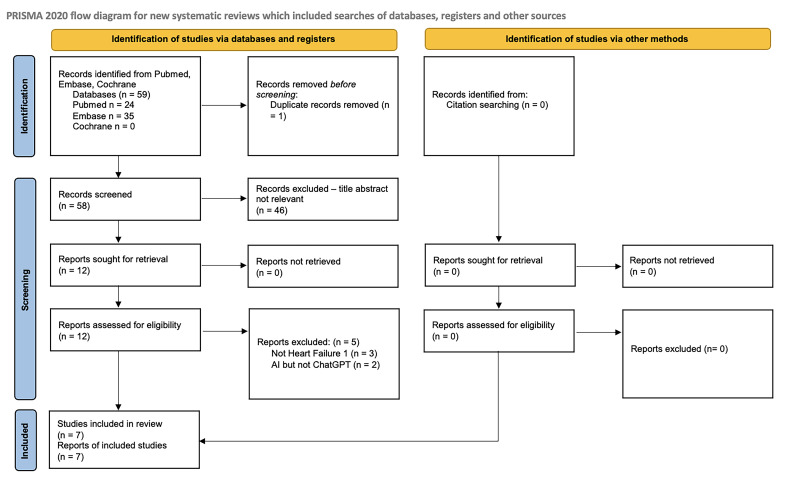
PRISMA 2020 flow diagram for new systematic reviews, which included searches of databases, registers, and other sources.

Our literature search identified a total of 59 records across PubMed, Embase, and Cochrane databases. After removing duplicates and screening for eligibility based on the inclusion and exclusion criteria, 7 studies were ultimately included in the review. The study selection process is summarized in Figure 1.

The key characteristics of the included studies, such as design, objectives, and methods, are outlined in Table 1. The evaluation methods, metrics used, and noted limitations for each study are summarized in Table 2.

## 4. Discussion

This systematic review synthesizes evidence from seven observational studies on ChatGPT’s applications in heart failure (HF), including applications relating to patient education, question-answering, readability enhancement, and symptom extraction from electronic health records (EHRs). These results align with emerging narrative reviews on ChatGPT in HF [16], which highlight its potential in personalized education and adherence support, but underscore gaps in systematic validation that this review addresses.

### 4.1. Key Findings and Thematic Synthesis

Thematically, the included studies cluster into three domains: patient education and question-answering [7,8,9,11,13], readability enhancement [9,12], and clinical documentation/symptom extraction [10]. Among the seven studies, the majority, *n* = 6, evaluated ChatGPT for patient benefit through education/question-answering, *n* = 5, and readability enhancement, *n* = 2, with overlap, while one focused on provider use via symptom extraction for workflow efficiency.

In education and question-answering, ChatGPT provided accurate, empathetic responses that could enhance patient understanding and self-care, with appropriateness rated 90–100% across HF topics like symptoms, lifestyle modifications, and medication management [7,8,11,13]. Dimitriadis et al. [7] reported 100% accuracy for ChatGPT-4 on 47 common HF questions, excelling in lifestyle advice, medication mechanisms, and symptom recognition. Accuracy was evaluated by two researchers who individually assessed the similarity, relevance, and reliability of responses based on the latest published guidelines for Heart Failure, with overall evaluation by the study’s primary supervisor [7]. Kozaily et al. [11] noted ChatGPT’s edge over Bard, Google’s AI chatbot, in diagnosis and prognosis but weaknesses in advanced areas like device therapy [11]. Bhupathi et al. [13] showed better accuracy and completeness for HF than rarer conditions like patent ductus arteriosus (PDA), attributed to greater training data, though using a standard measurement tool, the Patient Education Materials Assessment Tool, material understandability was higher for PDA than for HF information [13]. Anaya et al. [9] indicated comparable PEMAT readability but lower actionability, when compared with pre-existing patient educational materials from their institution. These comparisons highlight ChatGPT’s versatility but underscore limitations in advanced or less common topics.

The types of questions evaluated across the patient education and question-answering studies (*n* = 5) primarily encompassed common patient inquiries related to heart failure knowledge, management, and outcomes. Dimitriadis et al. [7] tested ChatGPT on 47 frequently asked questions derived from patient consultations, covering topics such as disease definition, symptom recognition, lifestyle modifications, medication mechanisms, familial/social support, and community resources. King et al. [8] assessed 107 questions curated from top cardiology institutions’ FAQs and patient education sections, categorized into “basic knowledge” covering topics such as heart failure symptoms and causes, management, and other miscellaneous topics. Anaya et al. [9] evaluated responses to frequently asked questions sourced from the ACC, AHA and HFSA, focusing on readability and actionability of educational content. Kozaily et al. [11] used 30 questions developed from online patient forums and physician expertise, addressing diagnosis, management, and prognosis. Bhupathi et al. [13] compared ChatGPT’s provision of information on heart failure versus patent ductus arteriosus through general queries about the conditions, emphasizing accuracy, completeness, and understandability using PEMAT scoring.

When asked to enhance readability, ChatGPT simplified complex texts but risked oversimplification. Anaya et al. [9] reported improvements in actionability through simplification of medical terminology, while King et al. [12] used GPT-4 to rephrase 143 institutional patient education materials (PEMs), reducing Flesch-Kincaid grade levels, maintaining 100% accuracy, and increasing comprehensiveness in 23% of cases. Anaya et al. [9] also found that ChatGPT-3 responses to HF frequently asked questions were at undergraduate levels, with higher difficult word percentages than ACC materials, yet achieved a PEMAT readability score exceeding the AHA’s. However, actionability was lower due to less effective prompts for behavior change [9]. In comparison, King et al. [12] lowered Flesch-Kincaid scores substantially for institutional HF PEMs but noted risks like loss of technical nuance. This domain contrasts with education and question-answering by focusing on text refinement rather than original content creation.

In symptom extraction, Workman et al. [10] used ChatGPT-4 to identify heart failure symptoms from simulated electronic health record notes. They applied a “zero-shot” approach, where the AI relies on its general knowledge without special training, and used prompt-engineering for better results. This achieved scores of 90% on precision and 100% on recall, outperforming traditional machine learning methods, which scored 65.5% (F1), without pre-labeled data [10]. Unlike the patient-focused tools for education and readability, this shifts to internal clinical tasks, using ChatGPT’s pre-trained knowledge without fine-tuning.

### 4.2. Implications for Heart Failure Care

ChatGPT addresses HF barriers like low health literacy and poor adherence [17] through conversational simplification [9], thereby building self-efficacy in weight and symptom monitoring, as well as fluid and dietary restriction [16]. Narrative reviews position it as a virtual assistant for diet, exercise, and coping [16,18], which could potentially reduce HF readmissions, which are strongly associated with low health literacy rates [17]. Adherence gains from reminders and explanations align with guideline emphasis on engagement [14]. EHR symptom extraction enables real-time phenotyping and wearable integration for proactive care [10,16]. Unlike the report generation capabilities being employed in radiology (50–100% accuracy [19]), the predominant benefits of ChatGPT in HF care may come in the form of patient-facing self-management tools.

ChatGPT could promote fairness by providing low-cost access to underserved groups [16], but biases such as its English focus might increase inequalities [16,19]. Adapting it for multiple languages is vital, given how social factors affect heart failure outcomes [16].

ChatGPT may contribute to adjustments in protocols for outpatient and inpatient heart failure care by incorporating AI-assisted tools into clinical workflows, potentially improving efficiency and personalization where supported by evidence. In outpatient settings, it could support patient self-management through conversational interfaces that provide education on medication adherence, symptom monitoring, and lifestyle modifications, with possible reductions in readmission rates via integrated reminders and query responses in mobile apps or telehealth systems, based on observed question-answering accuracy [7,8,9,11,13]. For inpatient care, ChatGPT may be able assist in protocols by facilitating symptom extraction from electronic health records, achieving up to 95% F1 scores [10], which might aid in risk stratification, team communication, and decision support for guideline-directed therapies, thereby helping to manage clinician workload and enable timely interventions. Any such integrations would necessitate careful protocol updates, including AI oversight, clinician training, and rigorous validation via randomized trials to confirm safety and effectiveness.

### 4.3. Drawbacks and Limitations of ChatGPT in HF Care

While ChatGPT has demonstrated reasonable performance in observational studies, several critical drawbacks must be considered. This is especially relevant for HF patients, whose care can be finely balanced, with even small changes in medications and behaviours potentially leading to decompensations. Current evidence suggests that care led by general physicians, and even general (non-HF specialist) cardiologists, in comparison with care lead by specialist HF clinicians, may lead to worse outcomes [20]. Although a 1.9% hallucination rate may appear modest [8], its potential to cause adverse outcomes, such as incorrect medication administration or nonadherence to guideline-directed lifestyle recommendations, could have significant clinical implications for heart failure patients. Two hallucinations were identified in King et al. [8] through independent grading of ChatGPT responses by two board-certified cardiologists, who evaluated for the presence of incorrect information. Responses were categorized as “completely correct,” “some correct and some incorrect,” or “completely incorrect,” with the 1.9% hallucination rate reflecting the two instances in GPT-3.5 that fell into the “some correct and some incorrect” category. In contrast, GPT-4 provided no inaccurate information in the study.

The hallucination in King et al. [8] resulted in a somewhat incorrect response regarding diagnostic risks, specifically GPT-3.5’s overgeneralization that heart failure inherently increases the risk of myocardial infarction without nuancing exceptions such as infiltrative cardiomyopathies. It underscores the broader risk of AI-generated misinformation leading to incorrect medication administration or nonadherence, potentially exacerbating decompensations in vulnerable HF patients. At a population level, this seemingly small rate could translate into substantial harm, as a 1.9% effect, whether beneficial or harmful, can affect thousands in large patient populations. For context, sacubitril/valsartan (Entresto) demonstrated an absolute risk reduction of 2.8% in all-cause mortality in the PARADIGM-HF trial, a finding that has been widely acclaimed and has markedly influenced contemporary heart failure guidelines and practice [21]. This 2.8% risk reduction seen in PARADIGM-HF is of a magnitude comparable to the 1.9% hallucination rate.

Furthermore, a key strength of ChatGPT lies in its broad accessibility and generally free availability. Much of its current accuracy derives from training on extensive internet datasets, which were largely unrestricted during its development. However, an increasing number of academic journals and professional societies are now implementing protections to prevent unauthorized use of their content for AI training, requiring licenses for such purposes. For instance, the European Society of Cardiology (ESC) has explicitly reserved rights under EU Directive 2019/790, opting out of text and data mining for AI development in their guidelines. This shift could profoundly impact the application of ChatGPT and other large language models in healthcare if access to the newest evidence-based content is no longer readily available for training. Consequently, patients relying on such models for health information may unknowingly receive compromised, outdated, or non-guideline-directed medical advice.

### 4.4. Strengths and Limitations of the Evidence

Strengths of the evidence include consistent accuracy across versions and validated readability gains, supported by expert grading. Limitations include observational designs, small samples, and lack of patient outcomes, restricting generalizability and mirroring radiology’s gaps [19]. Narrative reviews offer context but lack rigor; this review provides structured quality appraisal. Broader issues involve non-deterministic outputs (44.9% consistency [15]), outdated training risking misinformation [16], ethical concerns (privacy, liability [19]), data cut-offs, biases needing diverse sets, and hallucinations requiring tools like Retrieval-Augmented Generation [16].

### 4.5. Ethical Considerations

Ethical challenges in deploying ChatGPT for HF include liability, where responsibility for harmful advice could fall on developers, providers, or clinicians amid ambiguous regulations, potentially leading to legal disputes. Financial costs from lawsuits might burden institutions, deterring adoption without evolved insurance models. Contradictions with physician advice could cause confusion, non-adherence, or delays, exacerbating decompensations in HF patients, as seen in case reports of life-threatening misinformation [22]. This may depersonalize care, erode trust, and undermine the doctor-patient relationship. All of these questions are potential barriers to implementation which have not yet been adequately addressed in the Western world and beyond.

Broader ethical concerns in AI for healthcare include data privacy and security, as Large Language Models (LLMs) like ChatGPT process sensitive patient queries that could inadvertently breach General Data Protection Regulation (GDPR) if not properly anonymized or if data is used for model retraining without consent. Bias is another major issue: these models, trained on vast internet datasets, may perpetuate disparities by underperforming for underrepresented groups, such as in ethnic minorities or low-income HF patients, leading to inequitable care outcomes. Transparency poses challenges due to the “black-box” nature of LLMs, making it difficult for clinicians to understand or explain AI-generated advice, which contrasts with evidence-based medicine’s emphasis on verifiable reasoning. Informed consent is crucial; patients must be explicitly told they are interacting with an AI, not a human expert, to avoid deception and ensure autonomous decision-making. Finally, equity in access remains a concern; while ChatGPT’s low-cost availability is a strength, digital divides, such as a lack of internet or tech literacy in elderly HF populations, could widen health disparities unless mitigated through inclusive design and multilingual support.

### 4.6. Future Directions

Randomised trials evaluating the effect of ChatGPT versus standard education on patient outcomes including adherence and readmission rates are indicated. Regulatory frameworks, clinician training [23], and multilingual versions are essential for global HF management [24]. Longitudinal studies on engagement and cost-effectiveness, plus refined models for validity, bias, and ethics, will reinforce its utility [16].

## 5. Conclusions

In conclusion, ChatGPT shows considerable promise in improving heart failure management through enhanced patient education, accurate question-answering, improved readability of materials, and efficient symptom extraction. With accuracy rates exceeding 90% in most applications and significant readability gains, ChatGPT addresses critical barriers like low health literacy and adherence, potentially reducing HF’s global burden. However, risks of misinformation and ethical concerns necessitate cautious integration. Future research should prioritize randomized trials, real-world validations, and bias mitigation to harness ChatGPT’s full potential, ensuring equitable, safe, and effective AI-driven HF care.

## Figures and Tables

**Table 1:** Methodological characteristics of included studies.

Study	Primary Domain(s)	Sample Size	Intervention	Comparator	Key Outcomes
Dimitriadis et al. [7]	Patient Education and Question-Answering	47 questions	ChatGPT-4 *	None (observational)	100% accuracy in key areas such as lifestyle advice, medication mechanisms, and symptom management
King et al. (appropriateness) [8]	Patient Education and Question-Answering	107 questions	GPT-3.5 * and GPT-4	GPT-3.5 vs. GPT-4	98.1% appropriateness for GPT-3.5, with occasional hallucinations ^a^ (1.9%)
Anaya et al. [9]	Patient Education and Question-Answering; Readability Enhancement	12 questions	ChatGPT-3 *	Leading US institutes (ACC, AHA, HFSA)	78% actionability score, with competitive 75% PEMAT readability but lowest actionability among compared to materials
Workman et al. [10]	Clinical Documentation/Symptom Extraction	1999 snippets + 102 synthetic	ChatGPT-4 ZSL	ML and rule-based	95% F1 score for ZSL, outperforming baselines
Kozaily et al. [11]	Patient Education and Question-Answering	30 questions	ChatGPT-3.5 and Bard	ChatGPT vs. Bard	90% appropriateness for ChatGPT
King et al. (readability) [12]	Readability Enhancement	143 PEMs	GPT-4	Original materials	Improved readability to 6th-7th grade level
Bhupathi et al. [13]	Patient Education and Question-Answering	21 questions (10 HF, 11 PDA)	ChatGPT-3.5	AHA/ACC guidelines	Mean accuracy 5.4/6, 83.75% PEMAT-P understandability

^a^ Hallucinations refer to instances where the AI generates inaccurate or fabricated information. * ChatGPT-3: released in 2020 with 175 billion parameters, was a unimodal text-generation model proficient in producing human-like text but reported to struggle with coherence in long conversations, context retention, and safety, lacking extensive fine-tuning for dialogue and thus generating inconsistent or biased outputs; its training data cutoff was June 2020; GPT-3.5: an updated version, built on GPT-3’s conversational capabilities through better handling of natural language interactions, improved relevance and safety features to reduce harmful responses, and an expanded context window, with a data cutoff up to September 2021; GPT-4: launched in 2023 with a significantly larger undisclosed parameter count estimated at over 1 trillion, introduced multimodal processing through both text and images, superior reasoning and problem-solving abilities, and higher accuracy across complex tasks; OpenAI also reported reduced hallucination rates with GPT-4 and a much larger context window, with training data extending to April 2023, enabling more sophisticated applications in education, clinical decision-making, and content generation.

**Table 2:** Evaluation methods, metrics, and limitations of included studies.

Study	Expert Evaluation	ChatGPT Version	Evaluation Method	Metrics Used	Limitations Noted
Dimitriadis et al. [7]	2 cardiologist researchers independently assessed responses for similarity, relevance & reliability per guidelines; overall evaluation by primary supervisor.	GPT-4	Expert assessment by cardiologists on accuracy and comprehensiveness	Accuracy (%), comprehensiveness	Less comprehensive; limited to observational design
King et al. (appropriateness) [8]	2 board-certified cardiologists independently graded responses using predefined scale for accuracy & comprehensiveness; differences resolved by 3rd reviewer.	GPT-3.5 and GPT-4	Graded by board-certified cardiologists using 4-point scale (comprehensive to incorrect)	Appropriateness (%), reproducibility (%), comprehensive knowledge (%)	Occasional hallucinations * (1.9%); small sample of questions; no patient outcomes
Anaya et al. [9]	4 advanced HF attendings conducted blind assessment.	GPT-3	Blind assessment with PEMAT; readability calculators (Flesch-Kincaid, etc.)	PEMAT readability/actionability (%), grade level, word difficulty (%)	Longer responses at higher educational levels; lower actionability; observational only
Workman et al. [10]	2 clinicians independently annotated snippets; discrepancies resolved via discussion & consensus.	GPT-4	Zero-shot learning with prompt engineering; compared to ML/rule-based baselines	Precision (%), recall (%), F1 score (%)	Reliance on synthetic snippets; no real EHR validation; prompt sensitivity
Kozaily et al. [11]	2 HF experts independently & blindly evaluated answers for accuracy & consistency.	GPT-3.5	Expert evaluation by HF specialists; consistency across runs	Appropriateness (%), consistency (%)	Inadequacy in advanced topics; heterogeneity in comparators (Bard); small question set
King et al. (readability) [12]	1 board-certified cardiologist (not blinded) assessed accuracy & comprehensiveness.	GPT-4	Expert grading for accuracy/comprehensiveness; readability scores	Flesch-Kincaid grade level, accuracy (%), comprehensiveness increase (%)	Institutional materials bias; no long-term impact assessment
Bhupathi et al. [13]	2 independent evaluators assessed referring to AHA info; disparities cross-checked vs. ACC guidelines.	GPT-3.5	Likert scales for accuracy/completeness; PEMAT for understandability	Accuracy score (mean/6), completeness (mean/3), PEMAT understandability (%)	Slightly lower accuracy for rarer conditions; no RCTs; potential data abundance bias

American College of Cardiology (ACC); American Heart Association (AHA); Heart Failure Society of America (HFSA); Patient Education Materials Assessment Tool (PEMAT); Zero-Shot Learning (ZSL); Machine Learning (ML); F1 score (F1), and Generative Pre-trained Transformer (GPT); *: In the context of large language models like ChatGPT, hallucinations refer to instances where the AI generates plausible-sounding but factually incorrect, fabricated, or nonsensical information, often due to limitations in training data or pattern-matching without true understanding.

## Data Availability

The data analyzed in this systematic review are derived from publicly available studies cited in the references section. No new primary data were generated; all extracted data are contained within the article. Further inquiries can be directed to the corresponding author.
